
JOURNAL INFORMATION
==============================
NLM Title Abbreviation: ScientificWorldJournal
Journal ID: ScientificWorldJournal
NLM Journal ID: 101131163
Journal ID: TSWJ

The Scientific World Journal

ISSN: 2356-6140
EISSN: 1537-744X
Publisher: Wiley

ARTICLE INFORMATION
==============================
PMCID: PMC6084646
PMID: 30147605
DOI: 10.1100/tsw.2001.229
Article ID: 757241
Article version: 1
Subjects: Short Report

Cyclin-Dependent Kinase Inhibitors Induce Apoptosis in Plant Cells

Strnad M. 1 *
Havlia L. 1
Binarova P. 1
Kryscaron V. 1
Hanuscaron J. 1
Siglerova V. 1
Boumlgre L. 2
Heberle-Bors E. 2
1 Laboratory of Growth Regulators, Institute of Experimental Botany ASCR & Palacký University, Šlechtitelů 11, 783 71 Olomouc, Czech Republic
2 Vienna Biocenter, Institute of Microbiology and Genetics, Vienna, Austria
*M. Strnad: strnad@risc.upol.cz
Publication date: 2001
Electronic publication date: 2001 Feb 16
Volume: 1
First page: 126
Last page: 126
Copyright: Copyright © 2001 M. Strnad et al.
Copyright year: 2001
License: This is an open access article distributed under the Creative Commons Attribution License, which permits unrestricted use, distribution, and reproduction in any medium, provided the original work is properly cited.
License URL: https://creativecommons.org/licenses/by/3.0/

Funding: Ministry of Education, Youth and Sports MSM 153100008

==============================
Miami *Nature Biotechnology* Short Reports
*TheScientificWorld* (2001) 1 (S3), 126SR
ISSN 1532-2246; DOI 10.1100/tsw.2001.229

### CYCLIN-DEPENDENT KINASE INHIBITORS INDUCE APOPTOSIS IN PLANT CELLS

M. Strnad[1*], L. Havlíček[1], P. Binarová[1], V. Kryštof[1], J. Hanuš[1], V. Siglerová[1], L. Bögre[2], E. Heberle-Bors[2]

[1]Laboratory of Growth Regulators, Institute of Experimental Botany ASCR & Palacký University, Šlechtitelů 11, 783 71 Olomouc, Czech Republic; and [2]Vienna Biocenter, Institute of Microbiology and Genetics, Vienna, Austria
* strnad@risc.upol.cz

**INTRODUCTION.** Programmed cell death (PCD) appears to be a ubiquitous process which allows a multicellular organism to eliminate unwanted or defective cells by the process of cellular disintegration without inducing an inflammatory response. As compare to animal cells molecular and cellular mechanisms underlying PCD (or apoptosis) in plants were not characterised yet, although there are many data describing PCD during plant development or resulting form an interactions with environment (hypoxia, plant pathogen) (1). Here we collected the evidence that programmed cell death is induced with cdk inhibitors treatment also in plant cells.

**METHODS.** Seeds of field bean (*Vicia faba* L.) were germinated at 25°C in Hoagland solution. Seedlings with about 2-cm long main roots were treated with cdk inhibitor bohemin or roscovitin at concentration from 20-300 μM for various time periods of 2.612,24 and 48h. CDC-2 was detected with rabbit polyclonal Ab prepared against to a peptide (RITARGALEHEYFKDIK). Microtubular structures were detected with mouse monoclonal antibody DmlA (Sigma) against α-tubulin, or with rabbit affinity purified antibody against α,β-tubulin heterodimer. The γ-tubulin was detected with mouse monoclonal antibody TU-31, or with affinity purified rabbit polyclonal antibody (2 ).

**RESULTS.** The purine analogues bohemine and roscovitine were used to study the role on CDKs in cell cycle progression and microtubule organisation in *Vicia faba* root tip cells. Both drugs inhibited the activity of immunopurified *Vicia faba* and alfalfa CDC2-kinase. The transcript levels of an A- and B-type cyclin, as well as of the *cdc2* genes, declined in treated root tips, while the mRNA level of a D-type cyclin gene was not affected. An observed transient arrest at the G1/S and G2/M regulatory points indicated that inhibition of the CDC2-kinase had an effect on both transitions (see Fig. 1). In contrast to the regular bipolar spindle in untreated cells, in drug treated metaphase cells abnormally short and dense kinetochore microtubule fibres were observed. These microtubules were randomly arranged in the vicinity of the kinetochores and connected the chromosomes. Thus, the chromosomes were not aligned on the metaphase plate but were arranged in a circle, with kinetochores pointing inwards and chromosome arms pointing outwards. γ-Tubulin, which plays a role in microtubule nucleation, also localised to the centre on the monopolar spindle. The observed abnormalities in mitosis, after inhibition of CDC2-kinase by specific CDK drugs, suggest a role for this enzyme in regulating some of the steps leading to a bipolar spindle structure. These compounds also induce apoptosis of different plant cells *in vivo*.

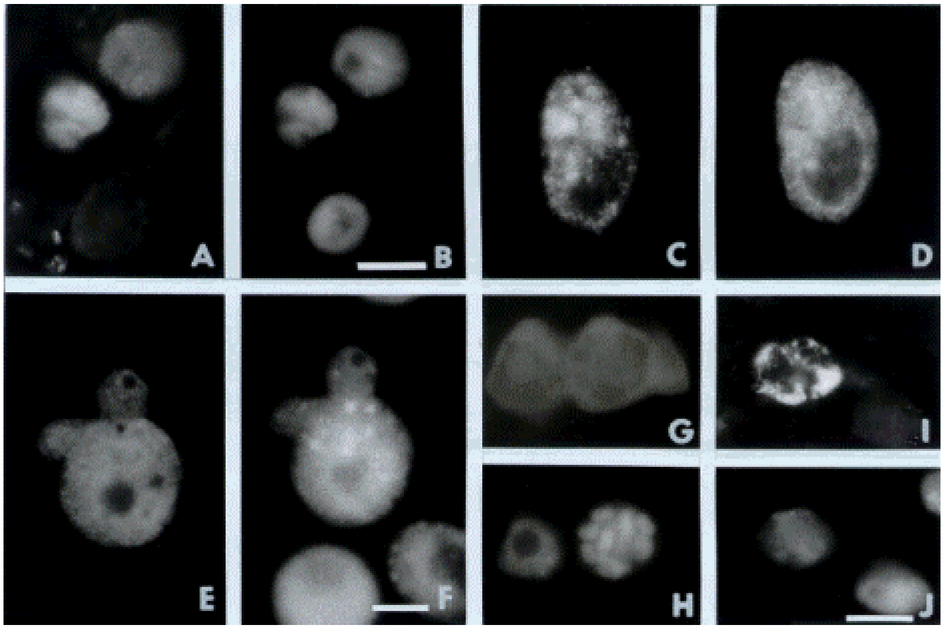

**Fig. 1.** Immunofluorescence in situ detection of DNA double strands breaks in cells of *V. faba.*

**DISCUSSION.** The doses of roscovitine used here to induce PCD in plant cells were several times higher than that reported as apoptosis inducing in animal cells (3). Lowered sensitivity of plant cells to roscovitin and bohemin treatment might be explained by different penetration or different metabolisms of the drugs, which are analogues of naturally occurring plant growth regulators from the group of cytokinins (4).

**ACKNOWLEDGEMENT.** This work is supported Czech Ministry of Education via grant MSM 153100008.

**REFERENCES.**

1. Pennell, R.I. and Lamb, C. (1997) Plant Cell 9, 1157-1168
2. Nováková, M., Dráberová, E., Schurman, W., Czihak, G., Viklický, V., Dráber, P. (1996) Cell Motil. Cytoskeleton 33, 38-51
3. Schutte, B., Nieland, L., Van Engeland, M., Henfling, M.E.R., Meier, L., and Ramaekers, C.S. (1997) Exp. Cell Res. 236, 4-15
4. Strnad, M. (1977) Physiol. Plant. 101, 674-688
